# Detection of Spiked *Fasciola hepatica* Eggs in Stool Specimens Using LAMP Technique

**Published:** 2019

**Authors:** Sahar GHODSIAN, Soheila ROUHANI, Shirzad FALLAHI, Seyyed Javad SEYYEDTABAEI, Niloofar TAGHIPOUR

**Affiliations:** 1. Department of Parasitology and Mycology, School of Medicine, Shahid Beheshti University of Medical Sciences, Tehran, Iran; 2. Department of Parasitology and Mycology, School of Medicine, Lorestan University of Medical Sciences, Khorramabad, Iran

**Keywords:** LAMP, *Fasciola hepatica*, Spiked egg

## Abstract

**Background:**

Fascioliasis is one of the most important food-borne worm disease caused by *Fasciola* sp. Parasitological diagnosis is more difficult due to the low parasite burden and a few eggs shedding of helminths. Therefore, it will be valuable to development of simple, fast and reliable diagnostic tests for detection of human and animal fascioliasis.

**Methods:**

Infected liver collected from abattoir in Tehran, Iran in 2017. *F. hepatica* eggs were detached from the uterus of worms under a stereo microscope. Various numbers of eggs were spiked to 200 mgr. of negative feces samples. DNA was extracted and then target regions (nuclear IGS) were amplified by LAMP assay using six primers. Fecal specimens without egg and DNA of other helminths were used as negative controls. *F. hepatica* sample which confirmed by morphologic criteria and PCR-RFLP was used as positive control.

**Results:**

LAMP products by using SYBR Green I could detect even a single egg in fecal samples which was visible by change of color from orange to green. There was no cross amplification by other helminths including; *Taenia saginata, Dicrosolium dendriticum* and *F. gigantica*.

**Conclusion:**

LAMP seems a rapid, sensitive, cost-effective technique for detection of human fascioliasis.

## Introduction

Fascioliasis is a zoonotic and serious disease caused by infection with the digenetic trematodes of the genus *Fasciola*. Fascioliasis is important in medicine and veterinary medicine. Parasite causes medical and economic losses in livestock products (1–3). This disease is more commonly found in America, Europe, Africa and Asia (1, 3–5). These parasites inhabit in the biliary system of the affected hosts. *F. hepatica* and *F. gigantica* typified based on morphological features (4, 6–9). *F. hepatica,* is a cosmopolitan parasite of cattle, sheep, buffaloes, goats as an intermediate host and human are accidental host (10).

Today, many diagnostic techniques have been used to detect *F. hepatica* infection in ruminants, including microscopic observation of worm eggs in feces, detection of antibodies in sera (11), coproantigen ELISA (cELISA) (12) in feces and several biochemical markers in the blood (13). In human, parasitological diagnosis is more difficult due to the low parasite burden and a few eggs shedding of parasite.

Recent molecular techniques such as PCR and loop mediated isothermal amplification are more rapid and accurate for detection of *Fasciola* sp. (14). PCR method has some limitations such as needs thermocycler and special detection devices. LAMP technique is a relatively new technology in which the reaction can run at a steady temperature and allows amplification of target nucleic acids with high specificity, sensitivity, rapidity, and precision (15, 16).

This assay uses a DNA polymerase, named Bst polymerase stranded replacement activity (17). This technique is highly specific due to a set of six primers, which binds to six distinct regions in the specific target DNA (17, 18). Simple visual monitoring of DNA amplification with the naked eye under a sunlight and UV light in the presence of SYBR Green I is one of the advantages of LAMP technique (16). The sequence selected in this study was the ribosomal intergenic spacer (IGS) sequence of *F. hepatica*. IGS sequence is located between the 3′ end of the 28S rRNA gene and the 5′ end of the 18S rRNA gene.

The aim of this study was to develop and evaluate rapid, sensitive, specific, and useful LAMP assay based on the IGS sequences for detection of spiked *F. hepatica* eggs in human feces.

## Materials and Methods

### Parasite and Spiking Protocol

*F. hepatica* samples were collected from the Maysam slaughterhouses, Tehran, Iran in 2017. The eggs were detached from the uterus of worms under a dissecting microscope. Eggs were accurately counted and variable number [1,1–5,10–15, 20–30, 50–60] of *F. hepatica* eggs were spiked to 200 mgr. of negative fecal samples were taken from under one-year-old kid (confirmed to be free of *F. hepatica* egg by the formalin-ether technique). Fecal specimens without egg and DNA of other helminths, including *T. saginata*, *F. gigantica* and *D. dendriticum* were used as a negative control. *F. hepatica* specimens previously confirmed by the authors of this paper by molecular methods and morphological standard were used as positive control (2, 3).

### PCR and LAMP assay

Genomic DNA was extracted from fecal samples containing spiked eggs by using the QIAamp DNA Stool Mini Kit (Qiagen) according to manufacturer's instructions. All experiments were done in triplicate. All extracted DNA samples were stored at −20 °C until further analysis. The *F. hepatica* specific primers used for the LAMP assay amplification were selected on the basis of a ribosomal intergenic spacer (IGS) sequences (16) (Table 1). The accuracy of the LAMP primers (F3 and B3) for the differentiation of *F. hepatica* were tested using other helminths control samples including *T. saginata, D. dendriticum* and *F. gigantica* by PCR assay. PCR reaction was performed in a 15 μL with 7.5μL master mix (Ampliqon, Denmark), 5.5 μL distilled water, 1μL primers (10 *pmol* of each primers), 1μL (1ng) DNA template. Initial denaturation was done at 94 °C for 5 min, followed by 30 cycles of denaturation (30 sec at 94 °C), annealing (30 sec at 52 °C), and extension (30 sec at 72 °C), and final extension for 7 min at 72 °C.

**Table 1:** LAMP primers sequences used for detection *F. hepatica* targeting the IGS region

***Primer***	***Length (bp)***	***Sequence (5′-3′)***
F3	20	CATTACCGACTCAGCTTGCA
B3	20	ACCAAACGTTCGGTTAAGGT
FIP	42	GCCGAATCAACCAGCCCTGAAAATGACGGTCCGGTATAGGTC
BIP	40	AGCGGATTCCAACTTCCATGGCACGCGACGCTCATGAGAT
FLP	20	GATGGCGCTGGAGCGTCGGA
BLP	18	CACCGTCCTGCTGTCTGG

The LAMP assay was conducted in a 25 μL volume with 2.5 μL of 10x Buff, 1.4 mM of dNTP, 8mM of Mgso4, 0.8 mM of Betaine, 40 pmol of each FIP and BIP primers, 20 pmol of each LF and LB primers, 5pmol of each F3 and B3 primers, 1 μL of target DNA, 8 Units of Bst DNA polymerase (New England. Biolabs), 4.5 μL of double distilled water. The reaction was incubated at 62 °C in a water bath for 1 hour. Amplifications were visually detected by adding 2 μl of 1:10 diluted 10000x concentration SYBR Green I dye (Invitrogen). We observed positive solution's color changed from orange to green. Additionally, the LAMP products were monitored using 1.5% agarose gel electrophoresis stained with safe stain.

## Results

**Fig. 1:**
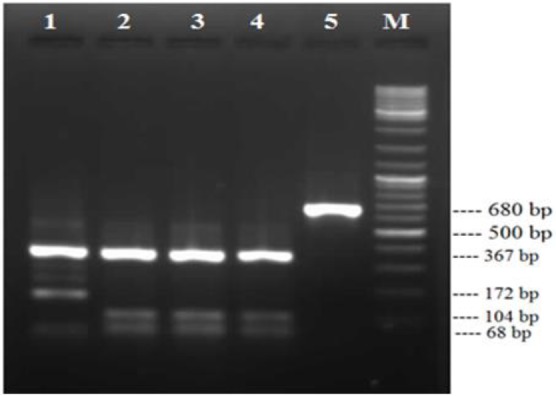
PCR-RFLP pattern of *Fasciola*, Lane1: ITS1 product of *F. gigantica* after digestion with RsaI enzyme, Lane2-4: ITS1 product of *F. hepatica* after digestion with enzyme, Lane 5: ITS1 product of *Fasciola* before digestion, Lane M: 100 bp DNA ladder

The *F. hepatica* samples, previously confirmed by the authors of this paper were used as positive control (Fig. 1).

**Fig. 2:**
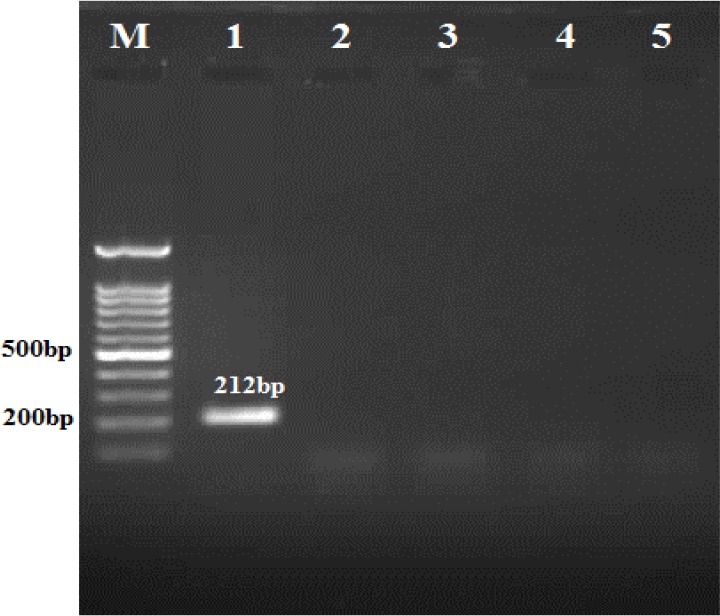
Specificity of PCR with the LAMP - primers F3 and B3, Lane M: 100 bp DNA ladder, Lane1: *F. hepatica*, Lane 2: *F.gigantica*. Lane 3: *D. dendiriticum,* Lane 4: *T. saginata,* Lane 5: negative control

The specificity of the LAMP primers for the identification and differentiation of *F. hepatica* was tested with other helminthes (control negative) (Fig. 2).

**Fig. 3:**
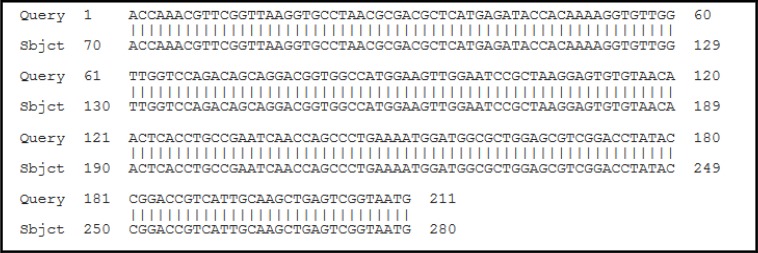
Sequence alignment of PCR band obtained in present study in comparison with database FhCM1-*F. hepatica* (GU903890)

Primers amplified a fragment of 212 bp; after sequencing, our results showed a 100% Identification, 99% Query coverage with the sequence (Fig. 3), consensus partial sequence of the 28S ribosomal RNA gene of *Fasciola* spp.

**Fig. 4:**
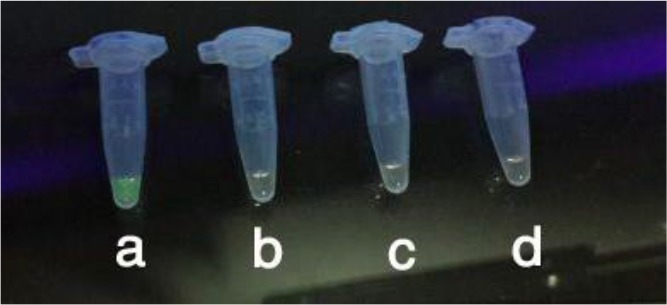
LAMP products under sunlight after the addition of SYBR Green I, a) *F. hepatica*, b) *D. dendiriticum* c) *T. saginata,* d) Negative control

In the following, the LAMP method was highly appropriate for the differentiation of the species. There was no cross-amplification by other helminths, including *T. saginata, D. dendriticum* and *F. gigantic* (Fig. 4).

**Fig. 5:**
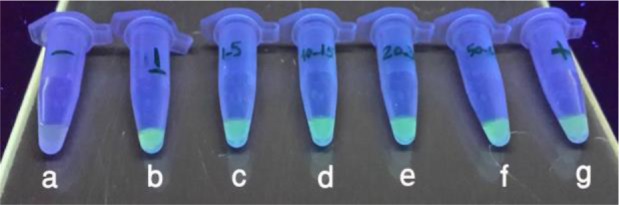
Sensitivity of the LAMP for detection of *F. hepatica* under UVlight after adding SYBR Green I, a) negative control, b) 1 egg, c) 1–5 eggs, d) 10–15 eggs, e) 20–30 eggs, f) 50–60 eggs, g) positive control.

To instate the detection limit of the *Fasciola* by the LAMP method, various numbers of *Fasciola hepatica* egg were tested. Results showed high sensitivity of LAMP, even in the low egg numbers. The Green fluorescent of the reaction tube was discernible by the UV light and sunlight at a low level of F*. hepatica* egg. LAMP could detect even a single egg in samples (Fig. 5).

**Fig. 6:**
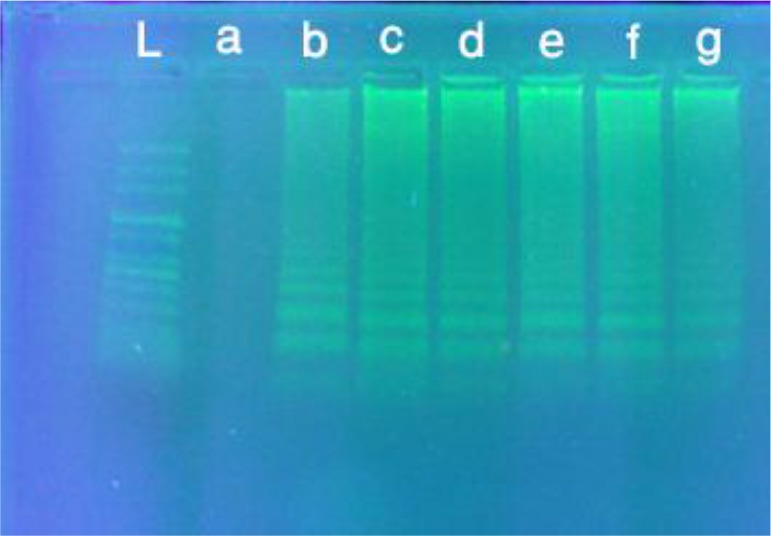
Agarose gel electrophoresis of LAMP products using various numbers of *F. hepatic*a egg, L) 100 bp DNA ladder, a) negative control, b)1 egg, c)1–5 eggs, d)10–15 eggs, e)20–30 eggs, f)50–60 eggs, g) Positive control

The amplification products were observed on 1.5% agarose gel as a ladder of multiple bands stained with safe stain (Fig. 6).

## Discussion

Fascioliasis is one of the zoonotic diseases with worldwide distribution caused by *F. hepatica* and *F. gigantica*. Fascioliasis has serious medical and economic impact in the world (1–3). *Fasciola* species colonize the biliary tract of humans and ruminants (1, 19). Early diagnosis of fascioliasis by quick, precise and sensitive way is very important to avoid the severity of the disease. The most common phenotypic approaches are often laborious and prolonged (16).

Despite the clinical symptoms, researchers are still wondering why most *Fasciola* eggs are not seen in common parasitological methods such as formalin-ether. The intensity of infestation and the number of eggs per gram of feces (epg) varies greatly in human fascioliasis. In most studies, low egg outputs, e.g. 1–2 eggs per gram of feces (epg) and 1–4 cross-amplification, have been mentioned (20, 21). In some studies, especially in high prevalence areas, an output of 440 epg in humans has been reported (22). Certainly, in low-intensity contamination, parasitological methods cannot detect *Fasciola* in infected individuals.

Recent molecular methods, including a LAMP technique, have greatly improved the quick diagnosis of many helminths such as *Fasciola* (16, 23, 24).

In a similar study to ours, the researchers added a variable number of *Echinococcus granulosus* eggs [1–2, 10, 25, 100] to the non-contaminated dog stool. The LAMP method was capable of detecting contamination in all groups, even 1–2 eggs (17).

A study was conducted to evaluate the LAMP method and the comparison of this technique with the standard microscopic method in diagnosis of *Opisthorchis viverrini* in Thailand (25). All samples that were positive by the microscopic method were reported positively by the LAMP assay. Five of the 13 samples were negative by microscopy, but positive by LAMP (38.5%) (25).

A study compared the LAMP method and PCR assay in diagnosis of *E. granulosus* in China. In this study, the positivity rates were 12.6% and 1.6% by the LAMP and PCR assay respectively. Moreover, the LAMP method could detect the presence of *E. granulsous* in infected dog, four days earlier than the PCR assay (26).

LAMP method is more rapid, accurate, sensitive and specific in comparison to other methods, such as PCR. Additionally, fecal inhibitors can impact the PCR results. The main problem of LAMP is that it cannot distinguish between the desired products and products of nonspecific amplification, thereby leading to false positives. There are different prevention methods of cross-contamination including, the use of UV (ultraviolet), LED (light-emitting diode) (27) and dual filter tips. Other ways to prevent contamination are closing reaction tube caps and putting them in plastic bags, cleaning environment with 75% ethyl alcohol (28) and paraffin oil added to the LAMP mixture (29).

Another benefit of LAMP technique is that the reaction can be performed on a water bath and it allows easy observation of the results, using UV light. If the LAMP materials hold at ambient temperature, these materials will still retain their quality. This feature could result in the usage of this method in the field settings or in limited laboratory facilities (30).

Another benefit of this method is the early diagnosis of infection compared to other methods. LAMP was used method to identify *E. multilocularis* in infected dogs. Dogs were experimentally infected with *E. multilocularis*. The LAMP method was able to identify and detect *E. multilocularis* DNA after 12 d post-infection. While the results of the PCR method were positive in 17 d after infection, and the first eggs were observed in the stool, 44 d after infection (31).

According to our literature review, this study is the first report of experimental usage of the LAMP method for detecting spiked *F. hepatica* eggs in feces. The *F. hepatica*-specific LAMP assays for IGS gene developed in this study is a flexible and quick method, which uses a water bath to maintain the temperature at 62 °C and obtains results within 60 min.

In this study, to prevent the risk of contamination, we autoclaved all equipment and place them under UV light for 20 min. After preparation of LAMP reaction, microtubes containing reaction solution were centrifuged at 12000 rpm for 3 min, and then were placed in −20 °C for 10 min. Finally, the microtubes were placed on the ice and added SYBR Green I dye. The method greatly prevented the contamination of environment and the subsequent reaction. This method of avoiding contamination was very beneficial.

Our results showed that LAMP assay has the potential to be practical in diagnosis and epidemiological researches. This method may be effective for the detection of disease in patients with suspected fascioliasis with the negative results of parasitological methods in endemic countries.

## Conclusion

This article was a preliminary study. In the future, the authors of this paper intend to investigate and compare this method with PCR and another technique in detection of spiked *F. hepatica* eggs in stool samples.

## References

[1] Mas-Coma S. Epidemiology of fascioliasis in human endemic areas. J Helminthol. 2005;79(3):207–16. 16153314 10.1079/joh2005296

[2] Reaghi S Haghighi A Harandi MF Molecular characterization of *Fasciola hepatica* and phylogenetic analysis based on mitochondrial (nicotiamide adenine dinucleotide dehydrogenase subunit I and cytochrome oxidase subunit I) genes from the North-East of Iran. Vet World. 2016;9(9):1034–1038. 27733809 10.14202/vetworld.2016.1034-1038 PMC5057026

[3] Rouhani S Raeghi S Spotin A. Spermatogenic and Phylo-molecular Characterizations of Isolated Fasciola Spp. From Cattle, North West Iran. Pak J Biol Sci. 2017;20(4):204–209. 29023077 10.3923/pjbs.2017.204.209

[4] Mas-Coma S Bargues MD Valero MA. Fascioliasis and other plant-borne trematode zoonoses. Int J Parasitol. 2005;35(11–12):1255–78. 16150452 10.1016/j.ijpara.2005.07.010

[5] Nguyen TG Van De N Vercruysse J Dorny P Le TH. Genotypic characterization and species identification of *Fasciola* spp. with implications regarding the isolates infecting goats in Vietnam. Exp Parasitol. 2009;123(4):354–61. 19733565 10.1016/j.exppara.2009.09.001

[6] Le TH De NV Agatsuma T Human fascioliasis and the presence of hybrid/introgressed forms of *Fasciola hepatica* and *Fasciola gigantica* in Vietnam. Int J Parasit. 2008 5;38(6):725–30. 10.1016/j.ijpara.2007.10.003 18031748

[7] Itagaki T Kikawa M Terasaki K Shibahara T Fukuda K. Molecular characterization of parthenogenic *Fasciola* sp. in Korea on the basis of DNA sequences of ribosomal ITS1 and mitochondrial NDI gene. J Vet Med Sci. 2005;67(11):1115–8. 16327222 10.1292/jvms.67.1115

[8] Itagaki T Sakaguchi K Terasaki K Occurrence of spermic diploid and aspermic triploid forms of *Fasciola* in Vietnam and their molecular characterization based on nuclear and mitochondrial DNA. Parasitol Int. 2009;58(1):81–5. 19087891 10.1016/j.parint.2008.11.003

[9] Rouhani S Raeghi S Mirahmadi H Harandi MF Haghighi A Spotin A. Identification of *Fasciola* spp. in the East of Iran, Based on the Spermatogenesis and Nuclear Ribosomal DNA (ITS1) and Mitochondrial (ND1) Genes. Arch Clin Infect Dis. 2017;12(2):e57283.

[10] Arifin MI Höglund J Novobilský A. Comparison of molecular and conventional methods for the diagnosis of *Fasciola hepatica* infection in the field. Vet Parasitol. 2016;232:8–11. 27890084 10.1016/j.vetpar.2016.11.003

[11] Mezo M González-Warleta M Carro C Ubeira FM. An ultrasensitive capture ELISA for detection of *Fasciola hepatica* coproantigens in sheep and cattle using a new monoclonal antibody (MM3). J Parasitol. 2004;90(4):845–52. 15357080 10.1645/GE-192R

[12] Brockwell YM Spithill TW Anderson GR Comparative kinetics of serological and coproantigen ELISA and faecal egg count in cattle experimentally infected with *Fasciola hepatica* and following treatment with triclabendazole. Vet Parasitol. 2013;196(3–4):417–26. 23643623 10.1016/j.vetpar.2013.04.012

[13] Fairweather I. Reducing the future threat from (liver) fluke: realistic prospect or quixotic fantasy? Vet Parasitol. 2011;180(1–2):133–43. 21703766 10.1016/j.vetpar.2011.05.034

[14] Föhse L Suffner J Suhre K High TCR diversity ensures optimal function and homeostasis of Foxp3+ regulatory T cells. Eur J Immunol. 2011 11;41(11):3101–13. 21932448 10.1002/eji.201141986

[15] Martinez-Valladares M Rojo-Vazquez FA. Loop-mediated isothermal amplification (LAMP) assay for the diagnosis of fasciolosis in sheep and its application under field conditions. Parasit Vectors. 2016 2 05;9:73. 26847130 10.1186/s13071-016-1355-2 PMC4743319

[16] Ai L Li C Elsheikha HM Rapid identification and differentiation of *Fasciola hepatica* and *Fasciola gigantica* by a loop-mediated isothermal amplification (LAMP) assay. Vet Parasitol. 2010;174(3–4):228–33. 20933335 10.1016/j.vetpar.2010.09.005

[17] Salant H Abbasi I Hamburger J. The development of a loop-mediated isothermal amplification method (LAMP) for *Echinococcus granulosus* [corrected] coprodetection. Am J Trop Med Hyg. 2012;87(5):883–7. 22987649 10.4269/ajtmh.2012.12-0184 PMC3516264

[18] Fernández-Soto P Gandasegui Arahuetes J Sánchez Hernández A A loop-mediated isothermal amplification (LAMP) assay for early detection of *Schistosoma mansoni* in stool samples: a diagnostic approach in a murine model. PLoS Negl Trop Dis. 2014;8(9):e3126. 10.1371/journal.pntd.0003126 PMC4154662 25187956

[19] Spithill TW Dalton JP. Progress in development of liver fluke vaccines. Parasitol Today. 1998;14(6):224–8. 17040765 10.1016/s0169-4758(98)01245-9

[20] Bendezú P Frame A Hillyer GV. Human fascioliasis in Corozal, Puerto Rico. J Parasitol. 1982;68(2):297–9. 7077460

[21] Knobloch J Delgado E Alvarez A Reymann U Bialek R. Human fascioliasis in Cajamarca/Peru. I. Diagnostic methods and treatment with praziquantel. Trop Med Parasitol. 1985;36(2):88–90. 4023557

[22] Mas-Coma MS Esteban JG Bargues MD. Epidemiology of human fascioliasis: a review and proposed new classification. Bull World Health Organ. 1999;77(4):340–6. 10327713 PMC2557647

[23] Magalhães KG Jannotti-Passos LK Caldeira RL Isolation and detection of *Fasciola hepatica* DNA in *Lymnaea viatrix* from formalin-fixed and paraffin-embedded tissues through multiplex-PCR. Vet Parasitol. 2008;152(3–4):333–8. 18243563 10.1016/j.vetpar.2007.12.019

[24] Cucher MA Carnevale S Prepelitchi L PCR diagnosis of *Fasciola hepatica* in field-collected *Lymnaea columella* and *Lymnaea viatrix* snails. Vet Parasitol. 2006;137(1–2):74–82. 16427203 10.1016/j.vetpar.2005.12.013

[25] Arimatsu Y Kaewkes S Laha T Sripa B. Specific diagnosis of *Opisthorchis viverrini* using loop-mediated isothermal amplification (LAMP) targeting parasite microsatellites. Acta Trop. 2015;141(Pt B):368–71. 25268466 10.1016/j.actatropica.2014.09.012 PMC4454772

[26] Ni XW McManus DP Lou ZZ A comparison of loop-mediated isothermal amplification (LAMP) with other surveillance tools for *Echinococcus granulosus* diagnosis in canine definitive hosts. PLoS One. 2014;9(7):e100877. 25007051 10.1371/journal.pone.0100877 PMC4089910

[27] Karthik K Rathore R Thomas P New closed tube loop mediated isothermal amplification assay for prevention of product cross-contamination. MethodsX. 2014;1:137–43. 26150945 10.1016/j.mex.2014.08.009 PMC4472950

[28] Zhou D Guo J Xu L Gao S Lin Q Wu Q Establishment and application of a loop-mediated isothermal amplification (LAMP) system for detection of cry1Ac transgenic sugarcane. Sci Rep. 2014 5 09;4:4912. 24810230 10.1038/srep04912 PMC4014978

[29] Zhang X Liao M Jiao P Development of a loop-mediated isothermal amplification assay for rapid detection of subgroup J avian leukosis virus. J Clin Microbiol. 2010;48(6):2116–21. 20375232 10.1128/JCM.02530-09 PMC2884476

[30] Thekisoe OM Bazie RS Coronel-Servian AM Stability of Loop-Mediated Isothermal Amplification (LAMP) reagents and its amplification efficiency on crude trypanosome DNA templates. J Vet Med Sci. 2009;71(4):471–5. 19420851 10.1292/jvms.71.471

[31] Ni X McManus DP Yan H Yang J Lou Z Li H Loop-mediated isothermal amplification (LAMP) assay for the identification of *Echinococcus multilocularis* infections in canine definitive hosts. Parasit Vectors. 2014 5 30;7:254. 24886279 10.1186/1756-3305-7-254 PMC4081488

